# Co-selective Pressure of Cadmium and Doxycycline on the Antibiotic and Heavy Metal Resistance Genes in Ditch Wetlands

**DOI:** 10.3389/fmicb.2022.820920

**Published:** 2022-02-18

**Authors:** Meng-Fei Yu, Bizhi Shu, Zhixuan Li, Guihua Liu, Wenzhi Liu, Yuyi Yang, Lin Ma

**Affiliations:** ^1^Hubei Provincial Key Laboratory for Protection and Application of Special Plants in Wuling Area, College of Life Sciences, South-Central University for Nationalities, Wuhan, China; ^2^Chinese Academy of Sciences Key Laboratory of Aquatic Botany and Watershed Ecology, Wuhan Botanical Garden, Chinese Academy of Sciences, Wuhan, China; ^3^College of Life Sciences, University of Chinese Academy of Sciences, Beijing, China; ^4^Hubei Key Laboratory of Wetland Evolution & Ecological Restoration, Wuhan Botanical Garden, Chinese Academy of Sciences, Wuhan, China; ^5^Center of Plant Ecology, Core Botanical Gardens, Chinese Academy of Sciences, Wuhan, China

**Keywords:** antibiotic resistance gene, co-selective pressure, doxycycline, cadmium, metal resistance gene

## Abstract

Abuse of heavy metals and antibiotics results in the dissemination of metal resistance genes (MRGs) and antibiotic resistance genes (ARGs). Ditch wetlands are important sinks for heavy metals and antibiotics. The relationships between bacterial communities and MRG/ARG dissemination under dual stresses of heavy metals and antibiotics remain unclear. The responses of MRGs and ARGs to the co-selective pressure of cadmium (Cd) and doxycycline (DC) in ditch wetlands were investigated after 7-day and 84-day exposures. In ecological ditches, residual rates of Cd and DC varied from 0.4 to –5.73% and 0 to –0.61%, respectively. The greatest total relative abundance of ARGs was observed in the Cd 5 mg L^–1^ + DC 50 mg L^–1^ group. A significant level of DC (50 mg L^–1^) significantly reduced the total relative abundances of MRGs at a concentration of 5 mg L^–1^ Cd stress. Redundancy analysis indicated that Cd and DC had strong positive effects on most ARGs and MRGs after a 7-day exposure. Meanwhile, the class 1 integron gene (*intI1*) exhibited strong positive correlations with most ARGs and cadmium resistance genes (*czcA*) after an 84-day exposure. Network analysis showed that *Acinetobacter* and *Pseudomonas* were the potential dominant host genera for ARGs and MRGs, and tetracycline resistance genes (*tetA*), *czcA*, and *intI1* shared the same potential host bacteria *Trichococcus* after an 84-day exposure.

## Introduction

Doxycycline (DC), a low-cost broad-spectrum antibiotic, is widely used as a medicine for disease prevention and treatment and as a feed additive for growth promotion in animal husbandry (Zhang et al., 2015). The majority of antibiotics are poorly absorbed by animals and released into the aquatic environment via urine and feces (Deng et al., 2019). In addition, the overuse of chemical fertilizers is an important cause of global heavy-metal environmental pollution, especially in China. This large-scale usage results in heavy metals, especially cadmium (Cd), being released from soils, thereby entering nearby water bodies through irrigation and rainwater (Martins et al., 2014). Hence, heavy metals and antibiotics often coexist in aquatic environments, resulting in more widespread and complex contamination due to their interactions (Bazzi et al., 2020).

Previous studies reported that some bacteria formed defense mechanisms against heavy metals and antibiotic contaminants. These mechanisms were associated with a class of coding genes named antibiotic resistance genes (ARGs) and heavy metal resistance genes (MRGs) (Yang et al., 2019; Wang et al., 2020). The coexistence of heavy metals and antibiotics would enhance their co-selection effects, especially when ARGs and MRGs are situated in the same mobile genetic elements (MGEs), such as the class 1 integron-integrase (*IntI1*) (Di Cesare et al., 2016). Meanwhile, ARGs and MRGs can be transferred among bacterial communities and other eukaryotic organisms, such as plants and humans, through horizontal gene transfer (HGT) (Yang et al., 2019). Moreover, MGEs can be activated, leading to increased HGT under lower concentrations of heavy metals/antibiotics or even some natural conditions (Ubeda et al., 2005). Together, the coexistence of heavy metals and antibiotics might exacerbate the dissemination of MRGs and ARGs in a water body, which will in turn lead to harmful effects on human health.

Recently, extensive investigations have been performed on the resistance genes’ changes under the combined stresses of heavy metals and antibiotics in biofilm wastewater treatment systems (Jang et al., 2018; Ma et al., 2019). Combined feeding of CuO nanoparticles and sulfamethoxazole led to the inhibition of the nitrification process and amplification of the sulfonamide resistance gene 3 (*sul3*).

and *sulA* (MRGs) expression in sequencing batch reactors (SBRs) (Zhang et al., 2020). In addition, Zn(II) and tetracycline co-selective pressure-induced enzymatic activities were involved in nitrification and denitrification processes; the abundance of ARGs (*tetA*, *tetM*, and *tetX*) and MRGs (*czcA*, *czcB*, and *czcC*) decreases in SBRs (Wang et al., 2020). These results implied that the variations in MRGs and ARGs could disturb the pollutant reduction in biofilm wastewater treatment systems by affecting microbial communities and metabolic activities. However, the combined impact imposed by the dual stresses of heavy metals and antibiotics on MRGs and ARGs have never been investigated in wetland systems. Ecological ditches are a type of wetland system that have a crucial role in receiving agricultural non-point source wastewater (Faust et al., 2018). The accumulation of heavy metals and antibiotics in ecological ditches may reduce their purification efficiency. Thus, discerning the dynamic changes in resistance genes induced by heavy metals and antibiotics is conducive to understanding bacterial defense mechanisms and their impact on pollutant removal performance in wetland systems under dual stresses of heavy metals and antibiotics.

In the current study, Cd and DC were used as hypothetical pollutants to evaluate their effects on the migration and conversion of heavy metals, antibiotics, and resistance genes in ecological ditches. This study aimed to elucidate: (1) the variations in Cd and DC contents in effluents under short-term and long-term compound pollution stresses; (2) acute and chronic effects of Cd and DC on the evolution of MRGs, ARGs, and MGEs in effluents; (3) the combined effects of Cd and DC on microbial community structures in ecological ditches; (4) the correlations among MRGs, ARGs, MGEs, Cd, and DC; and (5) the relationships among MRGs, ARGs, and MGEs, and their potential hosts using network analysis.

## Materials and Methods

### Construction of Ecological Ditches

Twenty-one small-scale ecological ditches with a pore volume of 20 L were built. Their dimensions were 40 cm × 20 cm × 70 cm (length × width × height) (Supplementary Figure 1). In each ecological ditch, there were two layers of substrates (gravel, 10 cm, 10–20 mm Φ; ceramsite, 40 cm, 2–4 mm Φ; porosity, 0.4). Outlets were located at 2 and 30 cm above the ditch bottom. These were mounted for drainage (depth of 2 cm) and water sampling (30 cm). In each microcosm, three strains of *Canna indica* L. of uniform size were planted and pre-cultivated for 60 days.

### Experimental Design

The growth and stabilization of microorganisms in ecological ditches lasted for 2 months. The sequencing batch experiments started on June 28, 2019 and contained 12 batches (84 days) with a hydraulic retention time (HRT) of 7 days. The composition of the synthetic wastewater was: 200 mg L^–1^ of chemical oxygen demand (COD), 30 mg L^–1^ of total nitrogen (TN), 2 mg L^–1^ of total phosphorus (TP), and 20 mg L^–1^ of ammonia nitrogen (NH_4_^+^-N). Ecological ditches were evenly divided into seven treatments as follows: without Cd or DC (T0, control), only 1.0 mg L^–1^ of DC (T1), only 0.5 mg L^–1^ of Cd (T2), 0.5 mg L^–1^ of Cd + 1 mg L^–1^ of DC (T3), 0.5 mg L^–1^ of Cd + 50 mg L^–1^ of DC (T4), 5 mg L^–1^ of Cd + 1 mg L^–1^ of DC (T5), and 5 mg L^–1^ of Cd + 50 mg L^–1^ of DC (T6). In the first batch (short-term, 0–7 days) and final batch (long-term, 77–84 days), 200 mL of water was taken at a center sampling pipe at 0, 1, 10, 24, 48, 72, 120, and 168 h. Water samples were only collected at 168 h (7 days) in the other 10 batches.

### Measurement of Water Quality Parameters

Water quality parameters, such as TN, NH_4_^+^-N, nitrate nitrogen (NO_3_^–^-N), nitrite nitrogen (NO_2_^–^-N), and TP, were determined as previously described (Ma et al., 2021). The COD was measured using a spectrophotometer (DRB 200, Hach, Ames, IA, United States). Changes in TN, NH_4_^+^-N, NO_3_^–^-N, TP, and COD effluent contents for the ecological ditches within 84 sampling days are presented in Supplementary Figure 2.

### Assays of Heavy Metals and Antibiotics in Water Samples

The Cd concentrations in both the influent and effluent for the 12 batches of these 21 ecological ditches were determined using atomic absorption spectrometry (Varian DUO, 240FS + 240Z, Agilent, Palo Alto, CA, United States) (He et al., 2017). The DC concentrations in both the influent and effluent of ecological ditches were analyzed (Wen et al., 2021). Briefly, 100 mL of water was collected and filtered using filtration containing Na_4_EDTA, enrichment using solid phase extraction with Waters’ Oasis HLB cartridges (6 mL, 200 mg, Waters Corporation, Milford, MA, United States), and the contents of DC ultra-performance liquid chromatography-mass spectrometry (UPLC-MSE) (Xevo TQ-S, Waters, Milford, CT, United States).

### Analysis of Antibiotic Resistance Genes, Metal Resistance Genes, Mobile Genetic Elements, and 16S rRNA Gene

Resistance gene abundances in water at short term (7 days) and long term (84 days) were measured using the real-time polymerase chain reaction (PCR) technology (Pu et al., 2018). Briefly, 500 mL of overlying water was filtered to extract the total DNA. The PCR system consisted of 2× TB Green Premix Ex Taq II 5 μL, each primer 0.4 μL, template DNA 1 μL, Rox reference dye 0.2 μL, and water 3 μL. RT-PCR reaction conditions were as follows: pre-denaturation for 30 s at 95°C, 40 cycles of 30 s denaturation at 95°C, 30 s annealing at 60°C, and 45 s elongation at 72°C, and a final extension at 72°C for 5 min. A total of 22 primer sets was used to analyze the relative abundances of ARGs, MRGs, and MGEs in water samples. These primer sets included 12 ARGs (tetracycline resistance genes, *tetA*, *tetB*, *tetC*, *tetE*, *tetG*, *tetM*, *tetO*, *tetQ*, *tetS*, *tetW*, *tetX*, and *tetY*), six MRGs (cadmium resistance genes, *czcA*, *czcB*, *czcC*, *czcD*, *czcR*, and *czcS*), three MGEs (*intI1*, *intI2*, and *tnpA*), and the 16S rRNA gene. The plasmids of all target genes were manufactured by the Sangon Biotech Company (Shanghai, China). The standard samples were diluted to yield a series of 10-fold concentrations and used to prepare the standard curves. The specificities of the qPCR products were analyzed by melt curve analysis, and amplification efficiencies ranged from 95 to 105% with R2 > 0.99. All data, including control plasmids, gene copy numbers, and calculation of absolute gene copy numbers were normalized to 16S rRNA abundance. Detailed information on the primers is listed in Supplementary Table 1.

### Determination of Bacterial Community Structure

The bacterial community structure of water samples at Days 7 and 84 of these 21 ecological ditches was analyzed using high-throughput sequencing technology (Ma et al., 2021). Briefly, the hypervariable regions V3-V4 of bacterial 16S rRNA were amplified by PCR using the primers 338F (5-ACTCCTACGGGAGGCAGCAG-3) and 518R (5-ATTACCGCGGCTGCTGG-3). Reaction systems consisted of 2× Mix 12.5 μL, each primer 0.5 μL, template DNA 1 μL, and water 10.5 μL. The amplification procedures were pre-denaturation for 3 min at 95°C, 35 cycles of denaturation at 95°C for 30 s, annealing at 55°C for 30 s, and extension at 72°C for 30 s, and a final extension at 72°C for 5 min. Subsequently, the sequencing of bacterial 16S rRNA was performed using the Illumina Hiseq 2500 platform (Illumina, San Diego, CA, United States) to generate 300 bp raw reads. The obtained raw reads were quality-filtered, demultiplexed using Trimmomatic under specific parameters of quantitative insights into microbial ecology (QIIME) and merged using FLASH.

### Statistical Analysis

Data are expressed as mean ± standard deviation (SD). One-way ANOVA with Tukey’s *post hoc* tests were employed to analyze the differences between Cd and DC contents, resistant genes, and the remarkably changed genera abundance among treatments. Cd and DC contents, relative abundance of relative ARG copies, and microorganisms in each treatment were plotted in Sigmaplot 18.0. Redundancy analysis (RDA) was performed to determine the correlations among Cd and DC, ARGs, MRGs, and MGEs in short-term and long-term treatments using Canoco software (version 5.0; Microcomputer, Ithaca, NY, United States). Linear regression was used to identify the relationship between ARGs and MGEs, and MRGs and MGEs in the short term (7 days) and long term (84 days). Network analysis of co-occurrence among microbiome and ARGs, MRGs, and MGEs in short-term and long-term treatments was conducted using Gephi software version 0.9.2 (Gephi, WebAtlas, Paris, France). All statistical analyses were performed using SPSS software (version 20.0, SPSS Inc., Chicago, IL, United States). Statistical significance was defined as *p* < 0.05.

## Results

### Dynamic Changes of Cadmium and Doxycycline Concentration in Ecological Ditches

**FIGURE 1:**
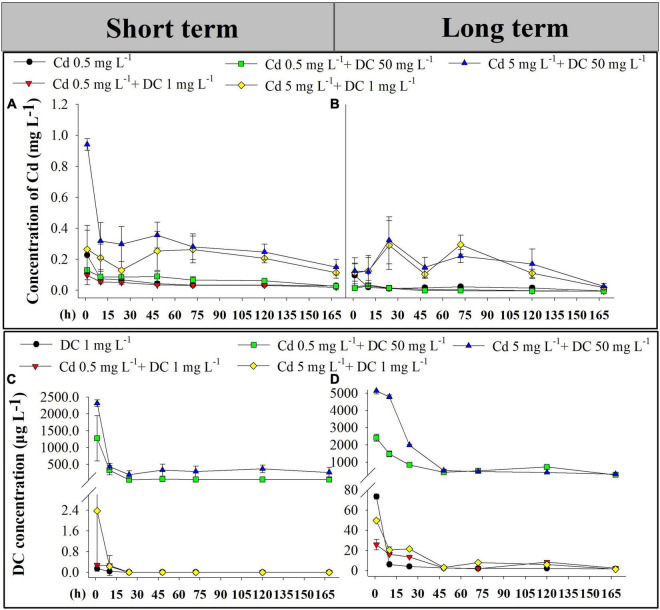
Cd and DC effluent concentrations in ecological ditches. Cd concentrations of Cd in short term (0–7 days) **(A)** and long term (77–84 days) **(B)**. DC concentrations in short-term observations (0–7 days) **(C)** and long-term observations (77–84 days) **(D)**. Cd, cadmium; DC, doxycycline.

The Cd and DC contents in the effluent from ecological ditches in short-term (0–7 days) and long-term (77–84 days) exposure experiments were investigated (Figure 1). No Cd or DC was detected in the control group. The concentrations of Cd and DC were near zero in the single DC group and the single Cd group, respectively. The residual rates of Cd and DC in the five corresponding ecological ditch groups varied from 0.4 to 5.73% and 0 to 0.61%, respectively.

As presented in Figures 1A,B, the concentrations of Cd in single Cd T2, low-Cd groups T3 and T4 decreased from 1 h to Day 1 and maintained relative low levels (about 0.06 mg L^–1^ for short term and 0.02 mg L^–1^ for long term) for the following 6 days. The Cd content in the high-Cd groups T5 and T6 showed a declining trend in short-term exposure experiments, while Cd contents in T5 and T6 systems fluctuated and abruptly increased at Days 1 and 3 during the long-term exposure experiments. In the short-term period, Cd content in T6 (Cd 5 mg L^–1^ + DC 50 mg L^–1^) was significantly greater than that in the other groups (*p* < 0.05).

The DC contents in T1 and T3–T6 groups decreased from 1 h to Days 1 (short term) and 2 (long term), and then remained constant until Day 7 (Figures 1C,D). With the addition of the same DC concentration, the DC contents in effluents under low-level Cd pressure (0.5 mg L^–1^) were less than those with high Cd levels (5 mg L^–1^) before Day 2 after short-term and long-term exposure. The residual concentration of DC in these five treatments after short-term exposure was significantly less than that of their corresponding counterparts after long-term exposure (*p* < 0.05).

### Variations in Resistance Genes of Ecological Ditches Under Dual Stresses

**FIGURE 2:**
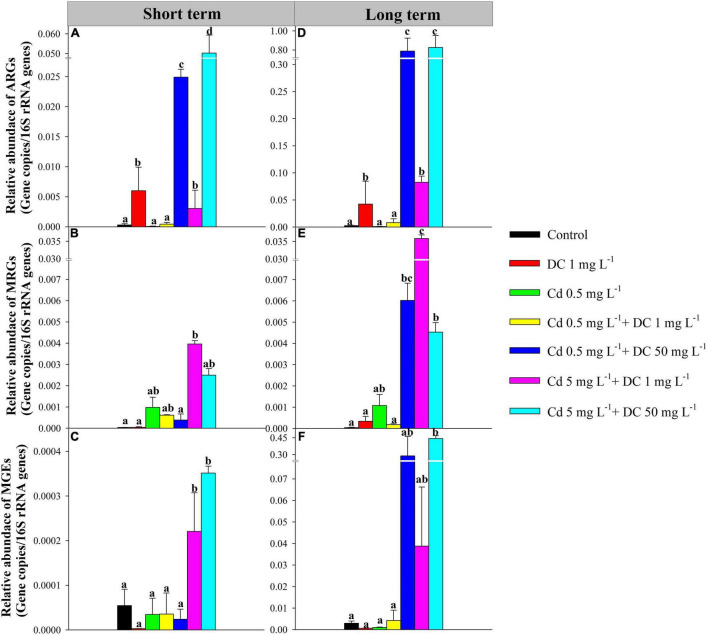
Relative abundances of detected ARGs, MRGs, and MGEs in effluent of ecological ditches in short-term [**(A–C)**, 7 days] and long-term observations [**(D–F)**, 84 days]. The different lowercase letters indicate significant differences (*p* < 0.05) among single and combined treatments of cadmium (Cd) and doxycycline (DC). ARGs, antibiotic resistance genes; MRGs, metal resistance genes; MGEs, mobile genetic elements.

The 12 tetracycline resistance genes, six Cd resistance genes, and three MGEs in ecological ditches after short-term and long-term exposure are shown in Figure 2.

#### Antibiotic Resistance Genes in the Effluent

**FIGURE 3:**
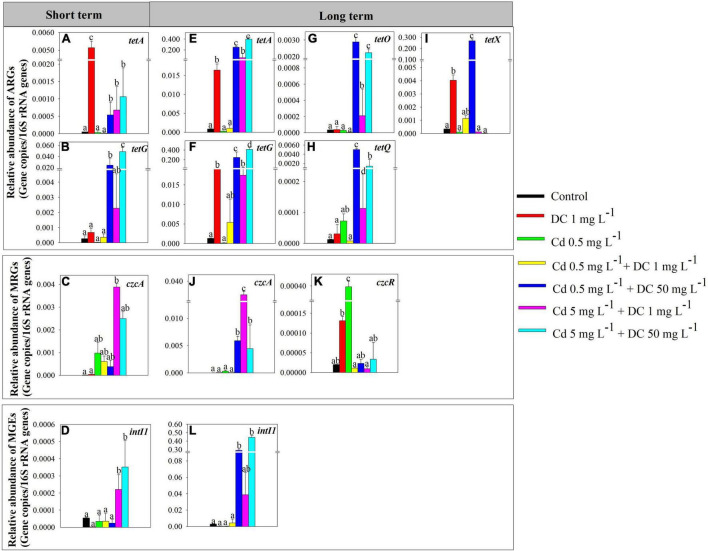
Significantly modified antibiotic resistance genes (ARGs), metal resistance genes (MRGs), and mobile genetic elements (MGEs) in effluent of ecological ditches in short-term [**(A–D)**, 7 days] and long-term [**(E–L)**, 84 days] observations. The different lowercase letters indicate significant differences (*p* < 0.05) among single and combined treatments of cadmium (Cd) and doxycycline (DC).

The high-Cd/DC group T6 had the highest relative abundance of total ARGs (5.02 × 10^–2^ copies/16S rRNA for short term, 8.26 × 10^–1^ copies/16S rRNA for long term), followed by the high DC group T4 (2.49 × 10^–2^ copies/16S rRNA for short term, 7.88 × 10^–1^ copies/16S rRNA for long term), and T5 (3.05 × 10^–3^ copies/16S rRNA and 8.26 × 10^–2^ for short term and long term, respectively) (Figures 2A,D). For the short-term period, *tetA* and *tetG* were the main components of ARGs in the ecological ditches. The relative abundance of *tetA* in the DC group T1 was significantly greater than that in the other six groups (*p* < 0.05) (Figure 3A), while the relative abundance of *tetG* in the high Cd/DC group T6 was significantly greater than that in the other six groups (*p* < 0.05) (Figure 3B). In the long-term period, *tetA*, *tetG, tetO, tetQ*, and *tetX* were the main components of ARGs in ecological ditches. The relative abundances of *tetA* and *tetG* in the high Cd/DC group T6 were significantly greater than those of the other treatments (*p* < 0.05) (Figures 3E,F); for both *tetQ* and *tetX*, relative abundances in the high DC group T4 were significantly greater than those of the other six groups (*p* < 0.05) (Figures 3G,I); for *tetO*, the relative abundance in the high DC groups T4 and T6 were significantly greater than those of the other five groups (*p* < 0.05) (Figure 3H).

#### Metal Resistance Genes in the Effluent

For the short-term period, the high Cd group T5 had the highest relative abundance of total MRGs (3.96 × 10^–3^ copies/16S rRNA), followed by T6 (2.49 × 10^–3^ copies/16S rRNA), and T2 (9.82 × 10^–4^ copies/16S rRNA) (Figure 2B). *czcA* (32.0–99.9%) was the main composition of MRGs in ecological ditches, and the relative abundance of *czcA* in the 5 mg L^–1^ + 1 mg L^–1^ Cd treatment was significantly greater than that of the other treatments (*p* < 0.05) (Figure 3C). Moreover, the highest relative abundance of total MRGs for the long-term period was also observed in the high Cd group T5 (3.59 × 10^–2^ copies/16S rRNA), followed by the high DC group T4 (6.02 × 10^–3^ copies/16S rRNA) and high Cd/DC group T6 (4.53 × 10^–3^ copies/16S rRNA) (Figure 3E). *czcA* and *czcR* were the main components of MRGs in ecological ditches, accounting for 15.5–99.7 and 0.03–49.6% of the total MRGs, respectively. The relative abundance of *czcA* in the high Cd group T5 was significantly greater than that in the other treatments (*p* < 0.05) (Figure 3J), while the highest relative abundance of *czcR* was found only in the Cd group T2.

#### Mobile Genetic Elements in the Effluent

Only the *intI1* was detected in all water samples, and the relative abundances of MGEs were 2.77 × 10^–6^–3.51 × 10^–4^ copies/16S rRNA, and 6.74 × 10^–4^–4.43 × 10^–1^ copies/16S rRNA in short-term and long-term periods, respectively. Moreover, the relative abundances of MGEs and *intI 1* in the high Cd groups T5 and T6 after short-term exposure were significantly greater than those of the other treatments (*p* < 0.05) (Figures 2C, 3D), while the relative abundance of MGEs and *intI 1* in the high DC groups T4 and T6 after long-term exposure were significantly greater than those of the other treatments (*p* < 0.05) (Figures 2F, 3L). Furthermore, there was a significant positive relationship between ARGs and MGEs in the long-term exposure experiment (R^2^ = 0.6364, *p* < 0.0001) (Supplementary Figure 3).

### Alterations in Microbial Community Under Dual Stresses

**FIGURE 4:**
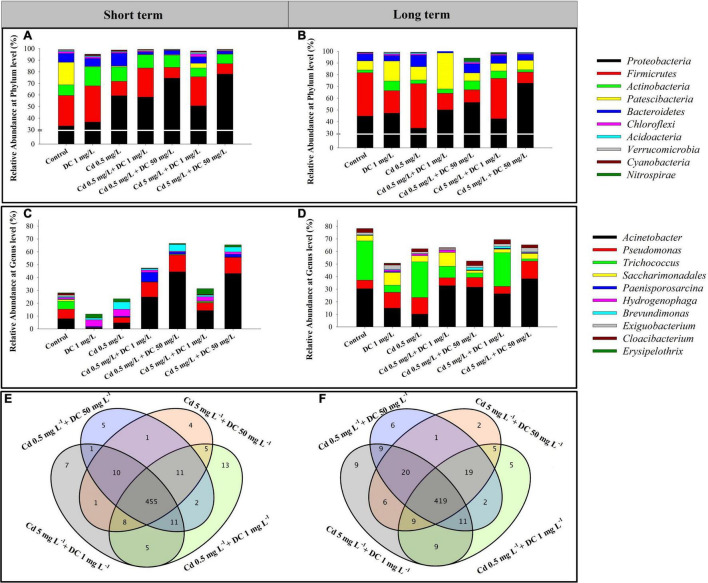
Relative abundance of bacterial community composition of the 10 most abundant bacteria at the phylum level **(A,B)**, and at the genus level **(C,D)** present in ecological ditch effluent in short-term (7 days) and long-term (84 days) observations. Venn diagram of the microbial community in combined pollutants treatments in **(E)** short-term (7 days) and **(F)** long-term (84 days) observations.

The relative abundances of the 10 most abundant bacteria at the phylum level were analyzed and are presented in Figure 4A (short term) and Figure 4B (long term). The predominant bacterial phyla in the combined pollutant treatments after short-term exposure were Proteobacteria (33.3–78.1%), Firmicutes (9.2–31.3%), Actinobacteria (7.5–16.4%), and Bacteroidetes (2.06–10.9%). In the combined pollutants treatments T3–T6, Firmicutes and Patescibacteria decreased remarkably by 1.21–17.43 and 15.27–19.16%, respectively, while Proteobacteria increased significantly by 17.35–44.82% compared with that of the control. For the long-term period, Proteobacteria (34.6–72.8%), Firmicutes (9.5–37.8%), Patescibacteria (6.4–30.7%), and Bacteroidetes (4.6–9.8%) were the principal phyla in the residuals. The relative abundance of Firmicutes declined by 26.23–27.47% compared to that noted in the control group.

In the short- and long-term periods, among the 539 and 532 assigned genera in 21 samples, 455 and 419 genera were shared by four dual-stress groups T3–T6, respectively (Figures 4E,F). The relative abundances of the top 10 genera were analyzed and are presented in Figure 4C (short term) and Figure 4D (long term). The total relative abundances of the top 10 bacterial genera in the short- and long-term periods accounted for more than 27 and 50% of the total genera in the four dual-stress groups T3–T6, respectively. The dominant genus in the combined pollutant treatments was *Acinetobacter*, belonging to the phylum Proteobacteria accounting for 8.35–44.46 and 26.42–38.36% in the short- and long-term periods, respectively. Moreover, six genera remarkably varied within the 7 days of Cd and DC addition (Supplementary Figure 4A). Among them, *Pseudomonas* in two high-DC groups T4 and T6 were greater than those of the control by factors of 1.77 and 1.69, respectively. In addition, *Acinetobacter* increased significantly to 44.46 and 26.32% in T4 and T6, compared with those of the control 7.96% (*p* < 0.05). In the long-term period, four bacterial genera *Acinetobacter*, *Pseudomonas*, *Trichococcus*, and *Saccharimonadales* varied significantly among the seven groups (Supplementary Figure 4B). *Acinetobacter* and *Pseudomonas* increased remarkably to 38.36 and 14.03% in the high Cd/DC group T6 compared with 30.42 and 6.77% in the control, respectively. Combined Cd and DC feeding significantly reduced the relative abundance of *Trichococcus* by 4.49–30.55% compared to that observed in the control.

### Correlations Among Resistance Genes, Heavy Metals, and Antibiotics

**FIGURE 5:**
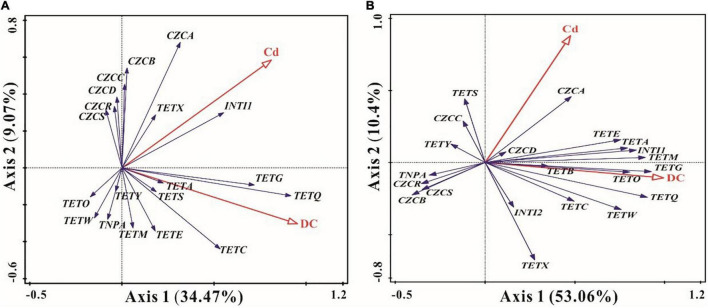
Redundancy analyses (RDA) of the relationships among antibiotic resistance genes (ARGs), metal resistance genes (MRGs), mobile genetic elements (MGEs), doxycycline (DC), and cadmium (Cd) in short-term [**(A)**, 7 days] and long-term [**(B)**, 84 days] observations. The angle between the arrows indicates the sign of the correlation between two entries.

Redundancy analysis was used to elucidate correlations among ARGs, MRGs, MGEs, DCs, and Cd. In the short term, the first two eigenvalues accounted for 34.47 and 9.07% of the total variation, respectively. The antibiotic DC and heavy metal Cd accounted for 43.5% of the total variations in ARGs, MRGs, and MGEs. Cd showed significant positive correlations with *tetA*, *tetG, tetQ, tetX, tetS*, and *tetC*, all detected MRGs, and *intI1*, while there were significant positive relationships between DC and ARGs, especially *tetG*, *tetQ*, *tetA*, *tetS, tetC*, and *tetE* (*p* < 0.05) (Figure 5A). Meanwhile, *intI1* had significant positive correlations with *tetX*, *tetG*, *tetQ*, *tetA*, and *czcA* in the short-term exposure experiments (*p* < 0.05) (Figure 5A).

For long-term periods, the first two eigenvalues accounted for 53.06 and 10.4% of the total variation, respectively. DC and Cd accounted for 63.1% of the total variation in ARG, MRG, and MGE expression. Cd and DC both had strong and positive effects on *tetA*, *tetE, tetM*, *tetG, tetB, tetO, czcA, czcD*, and *intI1* (*p* < 0.05) (Figure 5B). Meanwhile, *tetS*, *tetY*, and *czcC* were significantly positively associated with Cd, and significant positive relationships were observed between DC and *tetQ*, *tetC*, and *tetW* (*p* < 0.05) (Figure 5B). Moreover, *intI1* and *intI2* exhibited strong and positive effects on most ARGs (except for *tetS* and *tetY*), and a significant positive relationship between *intI1* and *czcA* was observed (*p* < 0.05) (Figure 5B). Furthermore, *czcA* and *czcD* genes were significantly positively correlated with most ARGs (except *tetY*) (Figure 5B).

### Relationships Between Resistance Genes and Microbial Community

**FIGURE 6:**
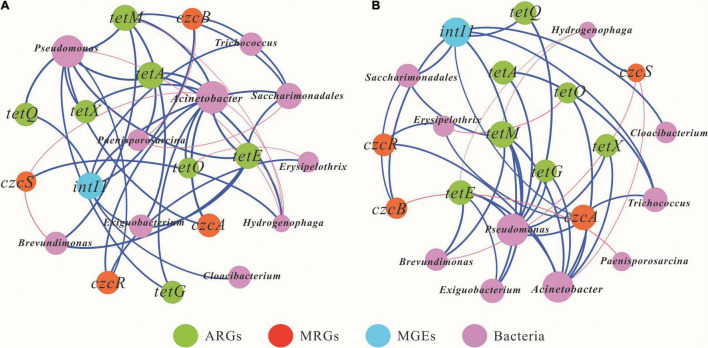
Co-occurrence network analysis presenting the correlations among antibiotic resistance genes (ARGs), metal resistance genes (MRGs), mobile genetic elements (MGEs), and the 10 most abundant bacterial genera in short term [**(A)**, 7 days] and long term [**(B)**, 84 days]. A connection represents a strong correlation based on Spearman’s correlation coefficient (*r* > 0.5, *p* < 0.05). An edge represents a strong correlation between two nodes, and the green edges and the red edges represent positive and negative relations between two nodes, respectively. The edge thickness is proportional to Spearman’s correlation coefficient (weight).

Co-occurrence network analysis was performed to elucidate the correlations among microbial community structure, resistance genes, and MGEs based on Spearman correlations, as shown in Figure 6. In the short term, the positive relationships among genera, ARGs, MRGs, and MGEs were 74.9% (Figure 6A). The bacterial genera *Acinetobacter* and *Pseudomonas* may be the most important potential hosts for ARGs. *Acinetobacter* showed positive correlations with six ARG subtypes, *tetA*, *tetE*, *tetM*, *tetX*, *tetO*, and *tetG*, while *Pseudomonas* was positively associated with *tetA*, *tetE*, *tetM*, *tetX*, *tetQ*, and *tetG*. Similarly, *Acinetobacter*, *Hydrogenophaga*, Saccharimonadales, and *Erysipelothrix* may be hosts for MRGs. *Acinetobacter* was the most important host for MRG and showed positive correlations with the two subtypes of MRGs, *czcA* and *czcR*. For the MGEs, the possible hosts for *intI1* were *Trichococcus*, *Saccharimonadales*, and *Cloacibacterium*.

As shown in Figure 6B, the positive relationships among genera and ARGs, MRGs, and MGEs in the long-term experiment was 58.9%. The bacterial genera *Acinetobacter*, *Pseudomonas*, *Trichococcus*, Saccharimonadales, *Brevundimonas*, and *Exiguobacterium* might be the main potential hosts for ARGs. *Pseudomonas* was the most important potential host for ARGs and showed significant positive correlations with six ARG subtypes: *tetA*, *tetE*, *tetG*, *tetM*, *tetQ*, and *tetX*. Similarly, the dominant host genera for MRGs were *Acinetobacter*, *Hydrogenophaga*, *Saccharimonadales*, *Trichococcus*, and *Erysipelothrix*. For example, *Acinetobacter* showed positive correlations with the two MRG subtypes, *czcA* and *czcR*. Significant negative correlations between genera and MRGs were also observed. *CzcS* showed significant negative correlations with *Acinetobacter* and *Brevundimonas*, whereas *czcB* was significantly negatively associated with *Paenisporosarcina*. For the MGEs, the dominant potential hosts for *intI1* may be *Cloacibacterium*, Saccharimonadales, and *Trichococcus*.

## Discussion

### Variations in Cadmium and Doxycycline Concentrations of Ecological Ditch Effluents

In the short-term and long-term experiments, Cd concentrations in the effluent of the five groups were less than 0.1 mg L^–1^ (Figure 1), suggesting that ecological ditches exhibited excellent Cd removal efficiencies. This was consistent with the results of previous studies showing that the contracted wetlands (CWs) removal efficiencies for Cd from industrial and mine wastewater were 91.9 and 94.0%, respectively (Yang et al., 2006). Meanwhile, Cd contents in the effluents of T5 and T6 (high Cd) were significantly greater than those in other groups after short-term exposure. This difference was not found in the long-term period (Figures 1A,B). This implies that wetlands could evolve a higher buffer capacity to cope with long-term changes in environmental conditions. This was confirmed by a previous study that reported that a 16-year-old constructed wetland maintained high stability for buffer capacity and heavy metal removal during the 10-year monitoring (Yang et al., 2006). Moreover, when the addition of DC concentration was consistent, DC concentrations in effluents less than 0.5 mg L^–1^ Cd stress were less than those at 5 mg L^–1^ Cd stress prior to Day 2 (Figure 1). This was ascribed to the low levels of Cd possibly enhancing DC sorption on ceramsite and plants, consequently resulting in higher DC removal efficiencies in ecological ditches. These results support the conclusion of Liang et al. (2018), who found that the addition of a low concentration of Cu (II) generally improved the reduction of SMX in CW microcosms (Liang et al., 2018). The complex formed between heavy metals and antibiotics may stimulate the antibiotic removal process (Wang J. et al., 2018). Furthermore, residual concentrations of DC in the five groups in the long-term period were significantly greater than those of their corresponding counterparts in the short-term period (Figure 1). Long-term antibiotic exposure induces the generation of antibiotic resistance genes, thereby causing the accumulation of antibiotics in CWs (Song et al., 2018).

### Evolutions of Antibiotic Resistance Genes, Metal Resistance Genes, and Mobile Genetic Elements at the Mixtures Stress of Cadmium and Doxycycline

The coexistence of Cd and DC in ecological ditches increases the occurrence and dissemination of ARGs and MRGs (Guo et al., 2018). The relative abundances of ARGs in T4 and T6 (high DC) were both significantly greater than those in the other groups; ARGs in the long-term period were more abundant than those of their corresponding counterparts in the short-term period (Figure 2). This indicated that ARGs increased in a time- and dose-dependent manner under the dual stresses of Cd and DC in ecological ditches. Similar results were observed in a previous study showing that ARG relative abundances in surface soils were significantly and positively associated with antibiotic concentrations. In addition, the relative abundances increased with time for all the groups in constructed wetlands (Huang X. et al., 2017). Meanwhile, in both short- and long-term exposure experiments, the relative abundances of ARGs in T5 (high Cd) were greater than those in T3 (low Cd), indicating that the addition of a higher Cd concentration increased the abundance of ARGs at 1 mg L^–1^DC stress (Figures 2A,D). This was principally because the complexes formed by heavy metals and antibiotics could enhance the expression of ARGs in microorganisms. This was confirmed by a previous study that reported that zinc could increase the relative abundance of ARGs by forming [Zn(CPH)_2_(OH)_2_] complexes (El-Said et al., 2009). *tetA* was the dominant subtype in ecological ditches (Figure 3A,C), which agrees with a previous study that reported that the relative abundance of *tetA* accounted for over 50% of the total ARGs in aquaculture pond samples (Huang L. et al., 2017). The relative abundance of *tetA* at 1 mg L^–1^ DC stress (T1) in the short-term experiment was significantly greater than that in the other six groups (Figure 3A), suggesting that *the tetA* gene was more sensitive to low DC levels alone rather than to the mixture stress. However, in the long-term period, the relative abundances of *tetA* in the high concentration Cd (T5) or DC (T4–T6) groups were significantly greater than those of other groups (Figure 3C), which might be due to Cd and DC dose-dependent synergistic effects on *tetA* in long-term experiments. The relative abundance of *tetG* in T1, T3, and T5 (low DC) was only detected in the long-term period (Figures 3B,F). This was mainly because the expression of *tetG* requires a higher threshold of DC (He et al., 2017).

For the MRGs, subtype *czcA* was the most dominant gene in ecological ditches (Figure 3). The incremental DC concentration at 5 mg L^–1^ Cd stress remarkably decreased the relative abundance of *czcA* and MRGs after short-term and long-term exposure (Figures 2B,D, 3C,J). This was mainly because high concentrations of antibiotics could stimulate the production and accumulation of extracellular polymeric substances (EPS), which in turn inhibited the expression of MRGs (Li et al., 2021). However, for the long-term period, the relative abundance of *czcA* in the Cd 5 mg L^–1^ + DC 1 mg L^–1^ treatment was relatively high, accompanied by higher relative abundances of *tetA*, *tetG*, and *intI1* (Figure 3). We hypothesized that the relative abundance of *czcA* increase was principally due to co-resistance via ARGs or enhancement of horizontal transfer potential via *intI1* (Shen et al., 2021). This speculation was confirmed by the results of RDA, which showed that *czcA* was significantly positively associated with *tetA*, *tetG*, and *intI1* (Figure 5).

### Variations in Bacterial Community Composition of Cadmium and Doxycycline

The dominant genus in the combined treatments was *Acinetobacter* in the short- and long-term periods, while the relative abundance of *Acinetobacter* in the Cd 5 mg L^–1^ + DC 50 mg L^–1^ treatment increased by 35.2 and 8%, respectively, of that noted in the control (Figure 4). This is mainly because *Acinetobacter* is known to have high levels of multiple heavy metals and antibiotic resistance (Dhakephalkar and Chopade, 1994). El-Sayed (2016) found that *Acinetobacter* isolated from industrial effluents exhibited multiple heavy metal and antibiotic resistance, with maximum tolerable Cd concentrations of 200 mg L^–1^ (El-Sayed, 2016). Moreover, the frequency of *Pseudomonas* slightly increased in T6 (high Cd and DC) after short-term and long-term exposure (Figure 5). This was consistent with a previous study showing that *Pseudomonas* sp. isolated from waste dumps of bauxite and magnesite mines demonstrated multiple metal- and antibiotic-resistant power (Narayanan and Natarajan, 2020). For the long-term period, the incremental addition of Cd resulted in a decrease in the relative abundance of *Pseudomonas* at 1 mg L^–1^ DC stress (Figure 5). This result supports the conclusion of Chen et al. (2015), who reported that the addition of arsenate, zinc, or copper significantly decreased the resistance of *Pseudomonas oryzihabitans* to tetracycline (Chen et al., 2015). Furthermore, the relative abundance of *Trichococcus* significantly increased in the long-term exposure experiment (Figure 5), suggesting a higher tolerance of *Trichococcus* to heavy metals. Qian et al. (2017) also found that in a bioreduction reactor, *Trichococcus* was the dominant fermenting genus under Cr (VI)-existing conditions (Qian et al., 2017).

### Relationships Between Resistance Genes, Heavy Metals, Antibiotics, and Microbial Community

#### Correlations Among Antibiotic Resistance Genes, Metal Resistance Genes, Mobile Genetic Elements, and Heavy Metals and Antibiotics

Class 1 Integron Gene was significantly and positively associated with *tetX*, *tetG*, *tetQ*, and *tetA* in the short term and most ARGs in the long-term period (Figure 5). This indicated that MGEs, especially *intI1*, played a vital role in the HGT of ARGs in ecological ditches. This was confirmed by a previous study showing that the addition of graphene oxide might decrease the abundance of ARGs by removing MGEs in swine manure with copper pollution during anaerobic digestion (Zhang et al., 2019). Meanwhile, a significant positive relationship between *intI1* and the dominant MRG subtype *czcA* was observed after short-term and long-term exposure (Figure 5), indicating that *czcA* had a higher transfer potential due to its close link with *intI1* (He et al., 2017). The presence of *intI1* was widely used as an indicator of HGT in prokaryotes (Aydin et al., 2015). Moreover, earlier studies reported that the heavy metals Cu and Zn were key factors influencing ARGs and MRG variation during aerobic composting (Guo et al., 2019). This was consistent with our findings that Cd had strong and positive effects on *tetA*, *tetG, tetQ, tetX, tetS, tetC*, and all detected MRGs for the short-term period, while most detected ARGs, *czcA*, *czcD*, and *czcC* genes were associated with Cd in long-term exposure experiments (Figure 5).

#### Co-occurrence of Antibiotic Resistance Genes, Metal Resistance Genes, Mobile Genetic Elements With the Bacterial Community

Consistent with earlier studies (Guo et al., 2019; Zhang et al., 2019), *Acinetobacter* and *Pseudomonas* were the main host potential bacteria for doxycycline resistance genes, where they were significantly positively related to six types of ARGs in the short term, and five and six types of ARGs after long-term exposure, respectively (Figure 6). Meanwhile, *Hydrogenophaga* might be a potential host for the MRG subtype *czcS* after both short-term and long-term exposure (Figure 6). This was consistent with results from a previous study reporting that *Hydrogenophaga* was significantly predominant in Hengshi sediments, with a higher abundance of As, Cu, and Zn resistance genes (Wang X. et al., 2018). Moreover, most of the ARGs and MRGs co-occurred in *Acinetobacter* and *Pseudomonas* (Figure 6). Previous studies have demonstrated that MRGs have stronger effects on driving variations in the abundance of ARGs (Cui et al., 2018). For the long-term period, *tetA*, *czcA*, and *intI1* were both positively related to *Trichococcus* (Figure 6B). It is well established that ARGs, MRGs, and MGEs share the same potential host bacteria, and MGEs may make important contributions to the evolution of ARGs and MRGs (Chen et al., 2016; Zhang et al., 2016). This was confirmed by our results showing that the high Cd T5 group had a higher relative abundance of *Trichococcus*, with more abundant *tetA* and *czcA* genes in the long-term exposure experiment (Figure 3 and Supplementary Figure 4). In addition, Saccharimonadales, *tetM*, *czcB*, and *intI1* were significantly correlated in the long term (Figure 6B). Arumugam et al. (2021) reported that phage and plasmid sequences presented in a short microbial genome from the order Saccharimonadales might mediate the horizontal transfer of resistance genes.

## Conclusion

Ecological ditches possessed sustained and stable capabilities in removing pollutants under the dual stresses of Cd and DC presence. After an 84-day exposure, the greatest total relative abundance of ARGs was detected with the addition of Cd 5 mg L^–1^ + DC 50 mg L^–1^; a 50 mg L^–1^ DC addition could cause a decrease in the total relative abundances of MRG under Cd stress. The evolution of ARGs and MRGs in ecological ditches relied on the concentration of antibiotics and heavy metals after a 7-day exposure; ARGs and MRGs shared the same potential host bacteria *Trichococcus*, their propagation and proliferation were strongly affected by co-resistance and promoted via *intI1* after the 84-day exposure. These findings highlight the different dissemination mechanisms of resistance genes in wetlands under co-selective pressure for different durations of exposure time. There is an urgent need to strengthen efficacious policies and technologies to remediate antibiotics and metal pollutants in water environments.

## Data Availability Statement

The datasets presented in this study can be found in online repositories. The names of the repository/repositories and accession number(s) can be found at: The National Genomics Data Center (NGDC), part of the China National Center for Bioinformation (CNCB); CRA005807.

## Author Contributions

M-FY: investigation and writing. ZL and BS: investigation. GL and WL: software, methodology, and funding acquisition. YY and LM: investigation, writing, and reviewing. All authors contributed to the article and approved the submitted version.

## Conflict of Interest

The authors declare that the research was conducted in the absence of any commercial or financial relationships that could be construed as a potential conflict of interest.

## Publisher’s Note

All claims expressed in this article are solely those of the authors and do not necessarily represent those of their affiliated organizations, or those of the publisher, the editors and the reviewers. Any product that may be evaluated in this article, or claim that may be made by its manufacturer, is not guaranteed or endorsed by the publisher.
