# Supplementary material for: Co-selective Pressure of Cadmium and Doxycycline on the Antibiotic and Heavy Metal Resistance Genes in Ditch Wetlands
Source: Front Microbiol. 2022 Feb 18;13:820920. doi: 10.3389/fmicb.2022.820920 (PMC8895241; doi:10.3389/fmicb.2022.820920)
Supplement: Supplementary file 1 [file Data_Sheet_1.docx]

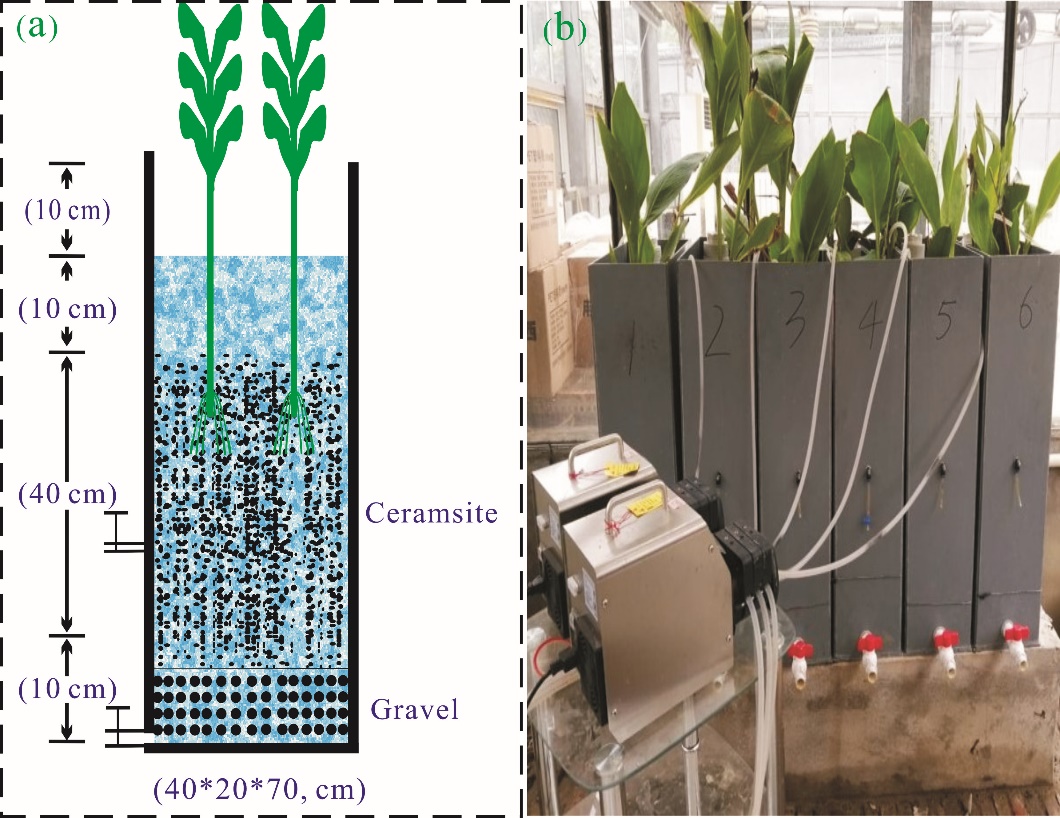


**Figure S1** A schematic diagram of the ecological ditch. (a) The simulation of the ecological ditch; (b) Six ecological ditches.


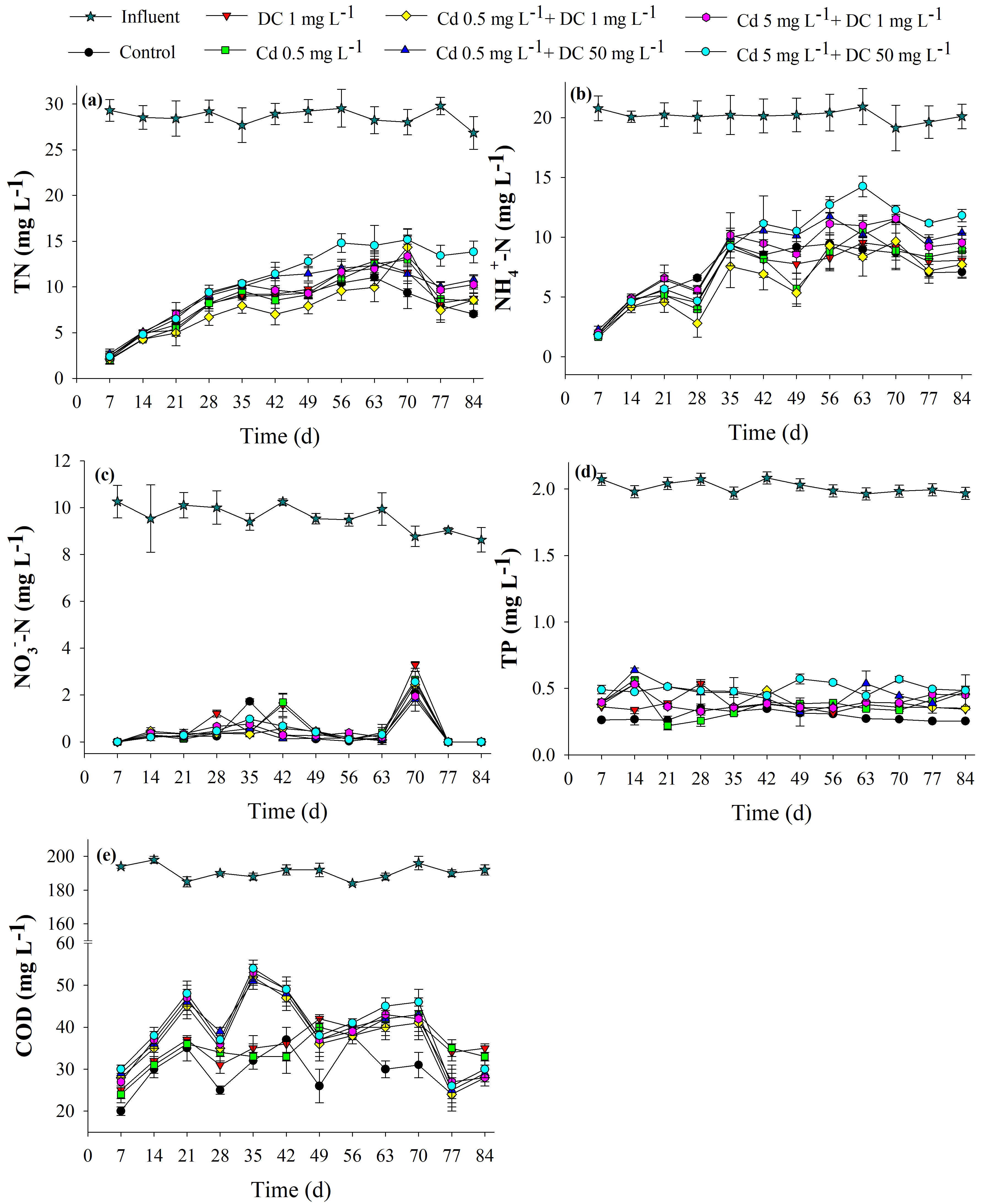


**Figure S2** Changes of TN (a), NH_4_^+^-N (b) , NO_3_^-^-N (c), TP (d), and COD (e) contents

in effluent of ecological ditches within 84 sampling days.


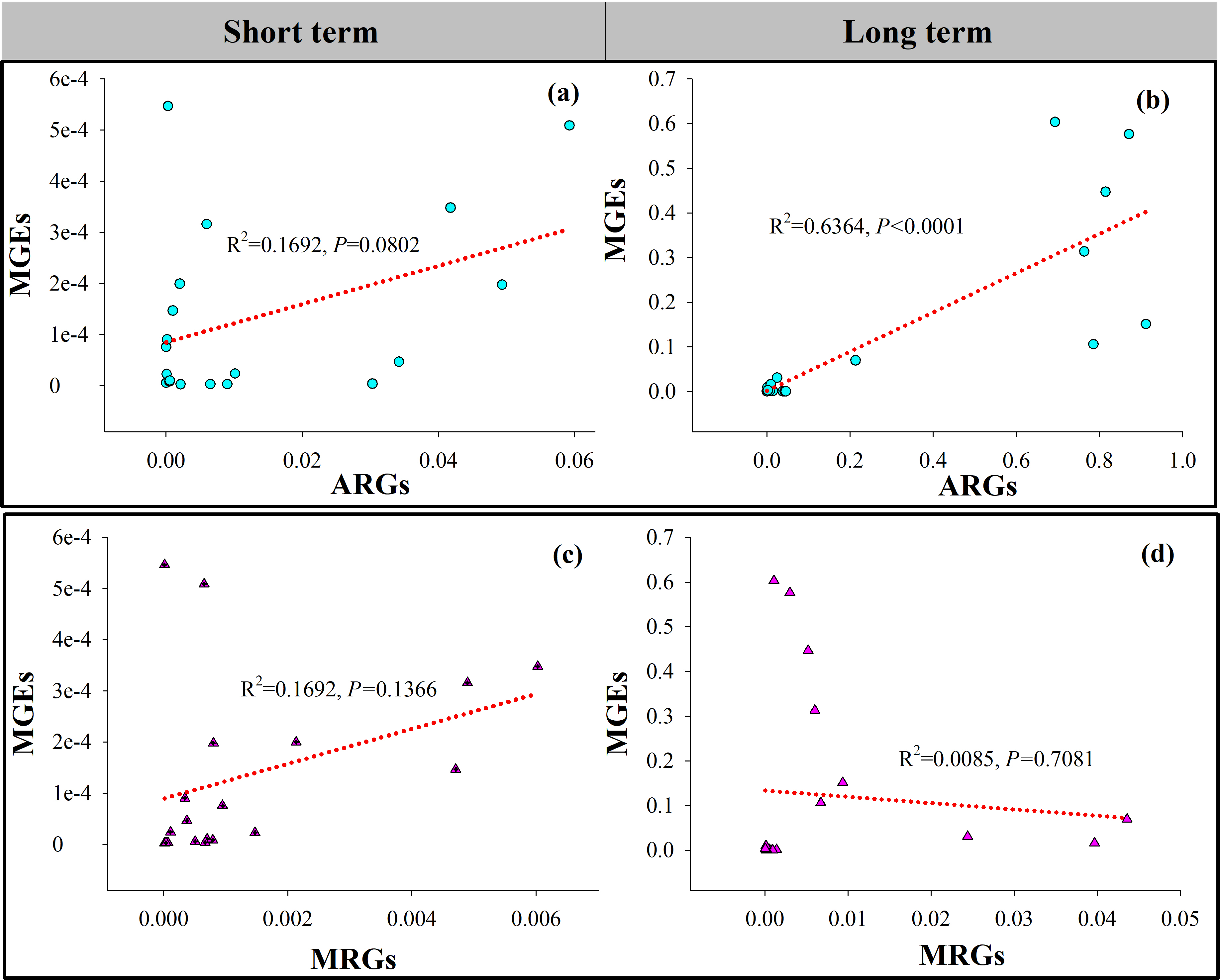


**Figure S3** Relationships between ARGs and MGEs (a and b), and MRGs and MGEs (c and d) in short-term (7d) and long term (84d).

**
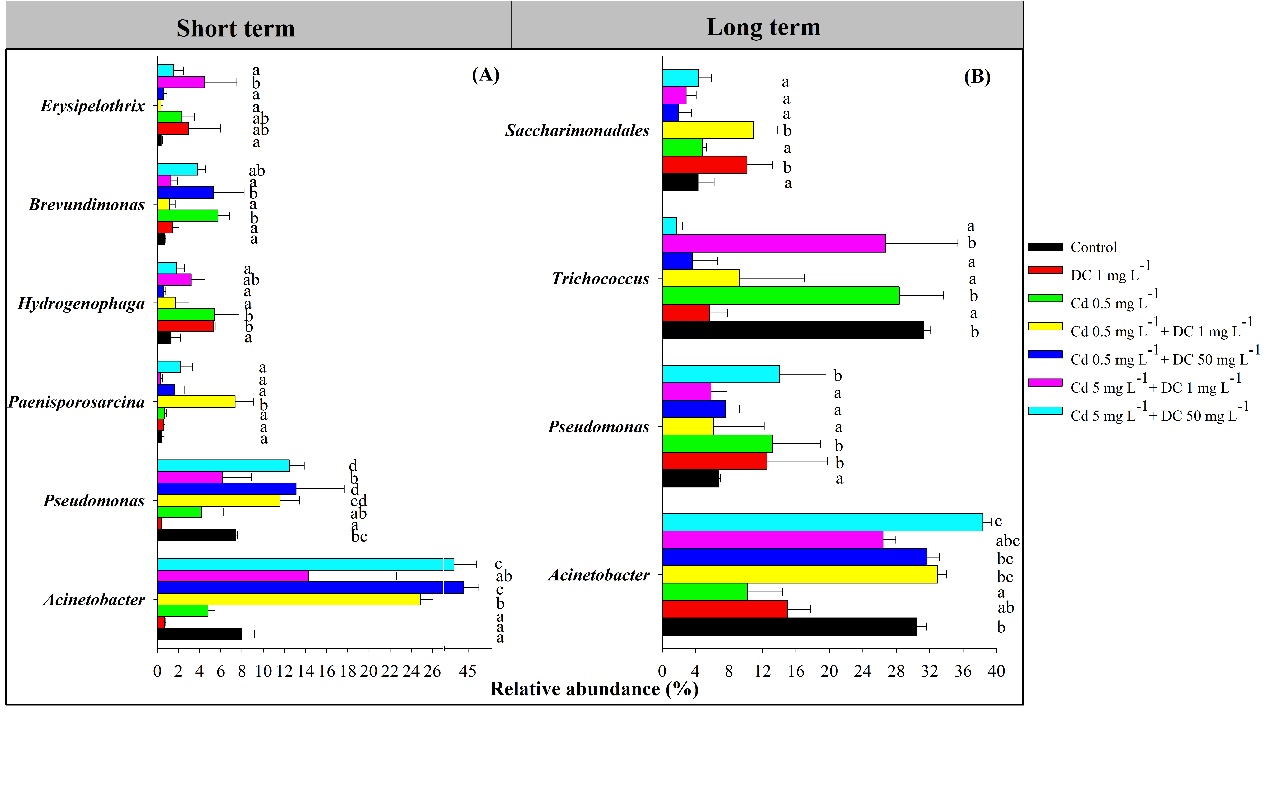
**

**Figure S4 The remarkably changed genera in effluent of ecological ditches** **in short-term (A, 7d) and long term (B, 84d). The different lowercase letters indicate significant differences (*p*<0.05) among single and combined treatments of Cd and DC.**

**Table S1 Primers of target genes used in qPCR analysis.**

| Target genes | Primer sequence (5’-3’) | Fragment size (bp) | References |
| --- | --- | --- | --- |
| *tet*A | F: GCTACATCCTGCTTGCCTTC | 210 | He et al., 2017 |
|  | R: CATAGATCGCCGTGAAGAGG |  |  |
| *tet*B | F: GAAGTAGGGGTTGAGACGCA | 136 | He et al., 2017 |
|  | R: TTTCGGTAAATAGCACCCACA |  |  |
| *tet*C | F: ACTACTGGGCTGCTTCCTAATG | 157 | He et al., 2017 |
|  | R: TCCTACGAGTTGCATGATAAAGA |  |  |
| tetE | F: GAAACCACATCCTCCATACGC | 153 | He et al., 2017 |
|  | R: AACACCGACCATTACGCCATC |  |  |
| tetG | F: TTATCGCCGCCGCCCTTC T | 133 | He et al., 2017 |
|  | R: TCATCCAGCCGTAACAGAAC |  |  |
| tetM | F: AGTGGAGAAATCCCTGCTCGGT | 149 | He et al., 2017 |
|  | R: TGACTATTTGGACGACGGGGCT |  |  |
| tetO | F: TTTCGGCTGCTTTCCCCTATCG | 133 | He et al., 2017 |
|  | R: TTGGAGCATCATGATACGCCCG |  |  |
| tetQ | F: AGAATCTGCTGTTTGCCAGTG | 169 | He et al., 2017 |
|  | R: CGGAGTGTCAATGATATTGCA |  |  |
| tetS | F: CAAAGTTGCTCCTTATATCCTGT | 176 | He et al., 2017 |
|  | R: TAGACAAGCCGTTGACCATC |  |  |
| tetW | F: GAGAGCCTGCTATATGCCAGC | 168 | He et al., 2017 |
|  | R: GGGCGTATCCACAATGTTAAC |  |  |
| tetX | F: GAAAGAGACAACGACCGAGAG | 131 | He et al., 2017 |
|  | R: ACACCCATTGGTAAGGCTAAG |  |  |
| tetY | F: GCTGATATTTGCGGGTTTCTA | 177 | He et al., 2017 |
|  | R: CGTCAAGCCTGTTAAAGTTCC |  |  |
| *czc*A | F:TCGACGGBGCCGTGGTSMTBGTCGAGAA | 232 | He et al., 2017 |
|  | R: GTVAWSGCCAKCGGVBGGAACA |  |  |
| *czc*B | F: GCAAGGAAGTCCTGACG | 202 | Zeng et al., 2012 |
|  | R: AGTTCGGATTTGAGGATGA |  |  |
| *czc*C | F: TGAACTTGCCCATCTCGAAG | 216 | Zeng et al., 2012 |
|  | R: GCAACCAGGGCAACATCTAC |  |  |
| *czc*D | F: TGAACGGCGGAGATACAAAG | 106 | Fierros-Romero  et al., 2019 |
|  | R: CTCCACCCGAAGAAGATGATAAG |  |  |
| *czc*R | F: GTCATCACCCGGACGCAGATCAT | 176 | Perron et al., 2004 |
|  | R: GTAGCCGACGCCGCGAATGGTAT |  |  |
| *czc*S | F: TACGCAGCTCTCGCAGTTCTCC | 168 | Perron et al., 2004 |
|  | R: TGTCCACCTGCACCAGGAACAGC |  |  |
| intl1 | F: CCTCCCGCACGATGATC | 280 | He et al., 2017 |
|  | R: TCCACGCATCGTCAGGC |  |  |
| intl2 | F: GTTATTTTATTGCTGGGATTAGGC | 166 | He et al., 2014 |
|  | R: TTTTACGCTGCTGTATGGTGC |  |  |
| tnpA | F: AATTGATGCGGACGGCTTAA | 216 | Zhu et al., 2013 |
|  | R: TCACCAAACTGTTTATGGAGTCGTT |  |  |
| 16S rRNA | F: CGGTGAATACGTTCYCGG | 142 | He et al., 2017 |
|  | R: GGHTACCTTGTTACGACTT |  |  |

Supplementary References for **Table S1**

Fierros-Romero G., M Gómez-Ramírez, Sharma A , et al. czcD gene from Bacillus megaterium and Microbacterium liquefaciens as a potential nickel-vanadium soil pollution biomarker[J]. Journal of Basic Microbiology, 2019, 60(1).

He, L., Liu, Y., Su, H., Zhao, J., Liu, S., Chen, J., Liu, W., Ying. G. Dissemination of antibiotic resistance genes in representative broiler feedlots environments: identification of indicator ARGs and correlations with environmental variables. Environmental Science Technology, 2014, 48(22): 13120-13129.

He, X., Xu, Y., Chen, J., Ling, J., Li, Y., Huang, L., et al. Evolution of corresponding resistance genes in the water of fish tanks with multiple stresses of antibiotics and heavy metals. Water Research. 2017. 124. 39-48

Perron, K., Caille, O., Rossier, C., van Delden, C., Dumas, J.-L., and Köhler, T. CzcR-CzcS, a Two-component System Involved in Heavy Metal and Carbapenem Resistance in Pseudomonas aeruginosa*. Journal of Biological Chemistry. 2004. 279:(10). 8761-8768

Zeng, X., Tang, J., Liu, X., Jiang, P., Response of *P. aeruginosa* E 1 Gene Expression to Cadmium Stress. Current Microbiology. 2012. 65, 799-804.

Zhu, Y., Johnson, et al. Diverse and abundant antibiotic resistance genes in Chinese swine farms[J]. Proceedings of the National Academy of Sciences of the United States of America, 2013, 110(9):3435-3440.
